# RiboSeq.Org: an integrated suite of resources for ribosome profiling data analysis and visualization

**DOI:** 10.1093/nar/gkae1020

**Published:** 2024-11-14

**Authors:** Jack A S Tierney, Michał I Świrski, Håkon Tjeldnes, Anmol M Kiran, Gionmattia Carancini, Stephen J Kiniry, Audrey M Michel, Joanna Kufel, Eivind Valen, Pavel V Baranov

**Affiliations:** School of Biochemistry and Cell Biology, University College Cork, Western Rd, Cork, T12 CY82, Ireland; SFI CRT in Genomics Data Science, University of Galway, University Rd, Galway, H91 TK33, Ireland; Institute of Genetics and Biotechnology, Faculty of Biology, University of Warsaw, ul. Pawińskiego 5A, Warsaw, 02-106, Poland; School of Biochemistry and Cell Biology, University College Cork, Western Rd, Cork, T12 CY82, Ireland; Computational Biology Unit, Department of Informatics, University of Bergen, Thormøhlensgate Bergen, 55N-5008, Norway; School of Biochemistry and Cell Biology, University College Cork, Western Rd, Cork, T12 CY82, Ireland; School of Biochemistry and Cell Biology, University College Cork, Western Rd, Cork, T12 CY82, Ireland; SFI CRT in Genomics Data Science, University of Galway, University Rd, Galway, H91 TK33, Ireland; EIRNA Bio, Food Science and Technology Building, 1 College Rd, Cork, T12 Y337, Ireland; EIRNA Bio, Food Science and Technology Building, 1 College Rd, Cork, T12 Y337, Ireland; Institute of Genetics and Biotechnology, Faculty of Biology, University of Warsaw, ul. Pawińskiego 5A, Warsaw, 02-106, Poland; Computational Biology Unit, Department of Informatics, University of Bergen, Thormøhlensgate Bergen, 55N-5008, Norway; Department of Biosciences, University of Oslo, Kristine Bonnevies hus, Blindernveien 31, 0731 Oslo, Norway; School of Biochemistry and Cell Biology, University College Cork, Western Rd, Cork, T12 CY82, Ireland

## Abstract

Ribosome profiling (Ribo-Seq) has revolutionised our understanding of translation, but the increasing complexity and volume of Ribo-Seq data present challenges for its reuse. Here, we formally introduce RiboSeq.Org, an integrated suite of resources designed to facilitate Ribo-Seq data analysis and visualisation within a web browser. RiboSeq.Org comprises several interconnected tools: GWIPS-viz for genome-wide visualisation, Trips-Viz for transcriptome-centric analysis, RiboGalaxy for data processing and the newly developed RiboSeq data portal (RDP) for centralised dataset identification and access. The RDP currently hosts preprocessed datasets corresponding to 14840 sequence libraries (samples) from 969 studies across 96 species, in various file formats along with standardised metadata. RiboSeq.Org addresses key challenges in Ribo-Seq data reuse through standardised sample preprocessing, semi-automated metadata curation and programmatic information access via a REST API and command-line utilities. RiboSeq.Org enhances the accessibility and utility of public Ribo-Seq data, enabling researchers to gain new insights into translational regulation and protein synthesis across diverse organisms and conditions. By providing these integrated, user-friendly resources, RiboSeq.Org aims to lower the barrier to reproducible research in the field of translatomics and promote more efficient utilisation of the wealth of available Ribo-Seq data.

## Graphical Abstract

**Graphical Abstract:**
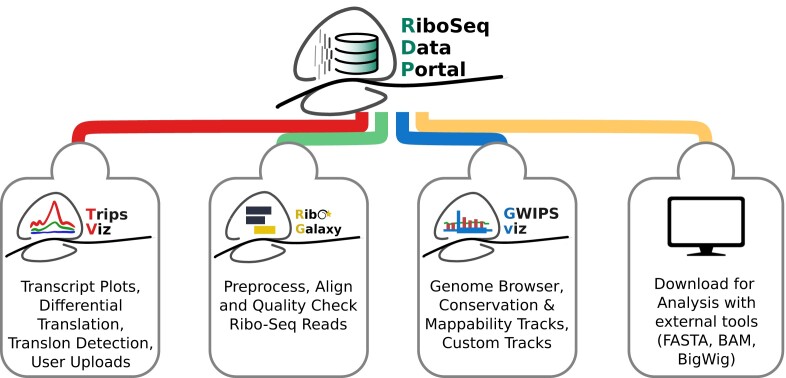

## Introduction

Ribosome profiling (Ribo-Seq) is a high-throughput method that enables the analysis of translation across the entire genome at sub-codon resolution. This is achieved through the arrest of ribosomes during translation. Following this arrest, RNA regions that are not protected by bound ribosomes are digested with ribonucleases. The remaining, ∼30 nucleotide-long, ribosome-protected fragments (RPFs or footprints) are then isolated and sequenced. The alignment of their sequences to a reference genome or transcriptome provides a global map of ribosomal occupancy that can be used to analyse translation and its dynamics (1–3).

Since the initial publication of this technique in 2009 (4), Ribo-Seq has evolved considerably, with several variants targeting ribosome complexes at different stages of translation. In addition to the most commonly used drug cycloheximide, which targets eukaryotic ribosomes in the pre-translocation conformation, several other drugs have been used to arrest elongating ribosomes in other conformations (5,6) as well as bacterial ribosomes (7,8). Furthermore, ribosomes can be preferentially arrested during the initiation of translation with lactimidomycin, harringtonine and retapamulin (for bacteria) (9–11) allowing mapping of translation initiation sites, while formaldehyde crosslinking can be used to arrest scanning complexes on eukaryotic mRNA (12). Further variations of these techniques enable the assessment of compartmentalised translation (13,14) and the dynamics of co-translational factors bound to the ribosomes (7,15,16). As a result, numerous applications of ribosome profiling data have emerged over the past decade.

Most commonly, ribosome profiling is used for investigating the regulation of gene expression at the level of translation (4,17,18) and the identification of translated regions outside annotated Coding Sequences (19–24).

**Figure 1.**
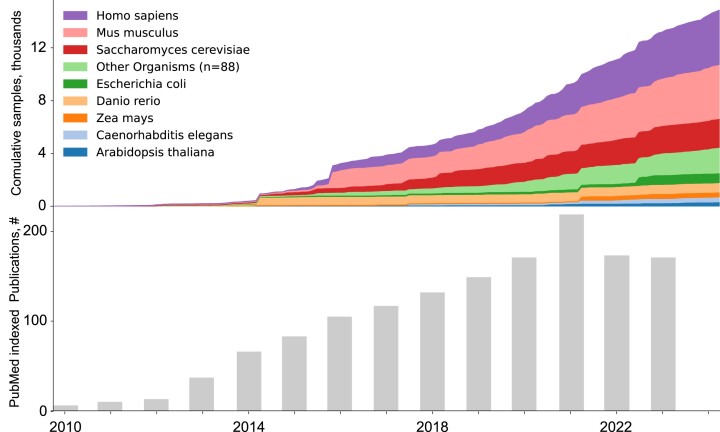
Growth of ribosome profiling data and literature. Top: Cumulative area plot illustrating the increasing number of publicly available ribosome profiling datasets across different organisms over time. Each curve represents a distinct organism, with the area under the curve filled to facilitate visual comparison. Bottom: Bar chart displaying the yearly trend in the number of publications related to ribosome profiling or Ribo-Seq, as indicated by the presence of these terms in the title or abstract of PubMed entries. The *y*-axis represents the count of relevant publications for each corresponding year on the *x*-axis. The decrease in the number of publications after 2021 likely reflects the drop in ribosome profiling being mentioned in the abstracts rather than a decrease in its use.

The popularity of Ribo-Seq has grown considerably as evidenced by the number of datasets deposited in public repositories such as the European Nucleotide Archive (ENA) (25) and the Sequence Read Archive (SRA) (26) (Figure 1). However, these data continue to be underutilised, due to the computational burden of data processing and inconsistencies in the accompanying metadata.

To address these challenges, numerous tools have been developed over the last decade to facilitate the translatomic research community’s access to preprocessed Ribo-Seq data (27). A prominent collection of processed ribosome profiling data (496 studies at the time of writing) is the ribosome profiling database (RPFdb v.3.0), which is a specialised database designed to store, organise and provide access to Ribo-Seq counts and translated ORF calls among other analysis outputs (28–30). TranslatomeDB, another web platform hosting Ribo-Seq data, contained 2453 Ribo-Seq datasets across 13 species at the time of publication in 2018 and focused on in-browser analysis (31). Other major ribosome profiling data re-processing initiatives resulting in populated web resources have been carried out primarily for the annotation of translated regions (20,32). While these are valuable resources, the lack of consistent metadata and lack of processed data in standard file formats limits potential for extensive reanalysis of the hosted data.

Over the last decade we have been developing a set of tools for ribosome profiling data analysis and visualisation GWIPS-viz (33,34), RiboGalaxy (35,36) and Trips-Viz (37,38) all of which are available through the Riboseq.Org portal. GWIPS-viz and Trips-Viz are populated with processed public Ribo-Seq datasets and support visualisation and analysis in a browser while RiboGalaxy enables end-to-end Ribo-Seq data processing in a web browser.

Despite these advancements, the independent development of various platforms has led to redundancies in preprocessing steps and inconsistencies in metadata handling. To address these issues and create a unified resource for the community, we aimed to standardise data processing across RiboSeq.Org resources and make the resulting processed data publicly available, thereby reducing the cost and effort of data reanalysis for the research community.

To this end we introduce the RiboSeq data portal (RDP). The RDP serves as a central repository of Ribo-Seq samples and studies, populated with standardised metadata and preprocessed data, addressing the key challenges of data reuse in the field of ribosome profiling. Unlike previous resources, RDP offers improved standardisation, more comprehensive metadata and seamless integration with analysis tools. In this paper, we describe the components of RiboSeq.Org, with a particular focus on the newly developed RDP, and demonstrate how this ecosystem can be used to gain new insights into translational regulation and protein synthesis across diverse biological contexts.

### RiboSeq.Org

RiboSeq.Org, with its suite of tools including GWIPS-viz, RiboGalaxy, Trips-Viz and now the RDP, together forms a comprehensive ecosystem for Ribo-Seq data analysis and visualisation. This integrated approach offers several key advantages:

- Standardisation of preprocessing steps across all RiboSeq.Org resources, reducing redundancy and discrepancies.
- Improved accessibility to high-quality, preprocessed Ribo-Seq data for researchers worldwide.
- Enhanced data interoperability between different tools, facilitating more comprehensive analyses.
- A centralised platform for standardised metadata, addressing inconsistencies in public repositories.

RiboSeq.Org has grown over the past decade to comprise several interconnected browser-based tools and resources that facilitate the processing, analysis and visualisation of ribosome profiling data. By providing these integrated resources, RiboSeq.Org aims to accelerate research in the field of translatomics, enabling researchers to fully utilise the wealth of publicly available Ribo-Seq data. Each component was developed to address different aspects of the Ribo-Seq data analysis, ranging from the processing of raw data to advanced data visualisation in genomic or transcriptomic contexts. Below we provide an overview of the features of existing RiboSeq.Org resources followed by a detailed description of the newly developed RDP.

GWIPS-viz (genome wide information on protein synthesis visualised) (33) is a genome browser tailored specifically for the visualisation of Ribo-Seq data alongside matched RNA-Seq data. It allows users to explore translation events across the entire genome guided by annotation, conservation and mappability browser tracks. GWIPS-viz is based on the UCSC genome browser (39) and presents data directly aligned to the genome allowing users to investigate the translation of both annotated and unannotated transcripts. GWIPS-viz serves as a valuable resource for researchers interested in a detailed view of translation at any location of a genome and the discovery of new translated regions as evidenced by its use to investigate translation in a large number of studies (40–45).

Trips-Viz, first published as a transcriptome browser (37), was subsequently extended to become a browser-based analysis platform (38). In contrast to GWIPS-viz, Trips-Viz is a transcriptome-centric browser where users can visualise ribosome footprints mapped directly to annotated RNA transcript sequences. Consequently, it is often possible to assign each aligned read to a specific reading frame based on the position of their inferred A-site relative to the transcript start site. This frame information is key for the accurate interpretation of overlapping translated regions (translons) (46) and may also be helpful for the identification of rare decoding mechanisms such as frameshifting or stop codon readthrough (47,48). In bacteria, the periodicity signal is generally weaker, due to the use of micrococcal nuclease for generation of bacterial data and footprint readlength variation caused by mRNA interactions with rRNA (49). Trips-Viz also can be used to calculate global parameters of the datasets, compare footprint density profiles of individual transcripts between sample sets, and carry out differential gene expression analysis among many other functionalities. The Trips-Viz platform has also been extensively used to investigate translation (50–52).

RiboGalaxy (36) provides a user-friendly, web-based environment for processing and analysing Ribo-Seq data using the Galaxy framework (53). It offers a comprehensive set of tools for Ribo-Seq data processing and analysis, with pre-configured workflows for standardised processing (e.g. for populating GWIPS-viz or Trips-Viz), while also supporting customisation of parameters to suit user specific needs. This platform empowers researchers, including those without extensive bioinformatics expertise, to perform sophisticated analyses of Ribo-Seq data using a graphical interface.

### RiboSeq data portal

The RDP is home to most of the publicly available ribosome profiling datasets preprocessed for downstream analysis accompanied by organised and manually curated information on the origins and experimental conditions. From this resource, a user can identify samples of interest, download processed data and associated metadata for their own downstream data analysis or analyse the data directly within their web browser using RiboSeq.Org tools (GWIPS-viz and Trips-Viz).

**Figure 2.**
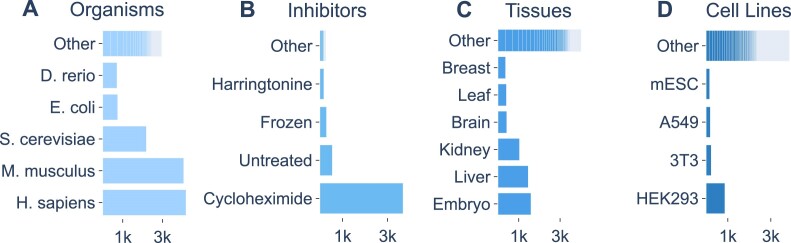
Metadata Landscape of RDP, A–D. Horizontal bar charts displaying the distribution of Organisms (**A**), inhibitors (**B**), tissues (**C**) and cell lines (**D**) present in the RDP Database. Each bar segment corresponds to a distinct category within the respective metadata field, with the segment width indicating the relative proportion of datasets associated with that category.

The current version (September 2024) of the Data Portal contains 14 840 samples. The metainformation for 4040 of which have been further validated manually by RiboSeq.Org contributors. These samples originated from a total of 969 Ribo-Seq studies across >96 different species/strains, see Figure 2 for more detail on the diversity of samples.

Each RDP sample has undergone a series of metadata curation steps using a controlled vocabulary consisting of a set of standard subject headings (e.g. cell line, tissue, inhibitor), paired with their own thesauri that enables the homogenisation of terms across studies. For instance, the terms ‘chx’, ‘cyclohex’ and ‘cycloheximide’ are converged to the term ‘cycloheximide’. This process resulted in the list of global metadata field names collapsing from a total of 813 entries to just 88. Of these, 31 core fields come directly from the metadata table downloaded from the SRA for each sample. Information is extracted from these core fields to identify Ribo-Seq samples and subsequently to populate other fields when they are not ascribed to their own field for that sample. An example of this is where the data depositors have not specified the cell line used in their own metadata column as it was specified in the sample’s name. By searching for keywords in core columns the correct cell line for this sample can be identified. However, priority is given to information specified explicitly in their own column. This standardised metadata enables coherent search and filter functionality across the database and is also made available for download in CSV format. Downloaded CSVs can then be used to specify the batches and conditions to be used in large-scale reanalysis.

Furthermore, sequence read files for each sample are obtained from the SRA, and are processed using standardised approaches (see below), ensuring that no additional variation is introduced due to variable data processing steps. The processing steps could be optimised for specific datasets to account for variations in cDNA libraries preparation, yielding a higher number of mapped reads. This can be done by designing a pipeline for a specific cDNA library construction including defining exact sequences of adaptors, UMIs (unique molecular identifiers, random nucleotide sequences added to alleviate ligation sequence biases and enable accounting for PCR bias) and so on. However, uniform processing enables comparative large-scale reprocessing. To enable uniformity, our method uses more tolerant alignment parameters that can handle various irregularities and idiosyncrasies, such as untemplated additions and inconsistent use of UMIs. These resulting processed files are available for download via the Data Portal. The primary file type made available is a collapsed read FASTA. In this format unique reads are stored once per sample and the number of occurrences of that read is stored in the sequence name. Other file types made available include BigWig and binary alignment maps (BAMs) along with the corresponding quality reports from tools such as FastQC and FASTP. The provision of these processed data files makes it possible for other researchers to carry out their own downstream analysis with varied degrees of flexibility without having to repeat redundant preprocessing steps when accessing the datasets from the SRA or ENA.

The development of the RDP has greatly improved the interoperability of decentralised web resources such as GWIPS-viz, Trips-Viz and RiboGalaxy allowing parallel exploration of translated regions at the genome and transcriptome levels with minimal effort.

#### Programmatic access facilitates large-scale analysis projects

For optimization of speed and storage, the RDP makes use of variants of standard file formats. Since the value of the data would be greatly diminished if manual or custom steps were required to integrate this data repository into an analysis workflow we developed RDP-Tools and the RDPs representational state transfer (REST) application programming interface (API) to automate and facilitate this integration. RDP-Tools is a Python-based command-line application that enables users to handle data files obtained from the RDP in their own workflow without the need for custom scripting. Primarily, it facilitates the collapsing and de-collapsing (inflating) of identical Ribo-Seq reads in various common file formats (FASTA, FASTQ and BAM).

Furthermore, RDP-Tools enables programmatic interaction with the REST API. Queries via the RDPs REST API return the requested metadata in JSON format. This may include download links that can easily be used to fetch the files from the portal as part of a data processing workflow. As a result, a user can identify, download and process Ribo-Seq samples for analysis without manual intervention.

#### Implementation

##### Ribo-Seq study and sample identification

Publicly available Ribo-Seq studies are identified *via* a multi-step process that runs monthly to ensure the database is up to date. First, a search is carried out against the NCBI-BioProject database for the keywords: ‘Ribosomal footprinting’ [all fields] or ‘ribosome footprinting’ [all fields] or ‘ribosome profiling’ [all fields] or ‘ribo-seq’ [all fields]. Then, the SRA Run meta information is downloaded for each study that was identified. This metadata is aggregated and a classification step is performed where individual runs are identified as Ribo-Seq or not Ribo-Seq. This classification is based on the presence or absence of the following terms in the sample name or sample title: ‘ribo’, ‘footprint’, ‘RPF’, ‘RFP’, ‘80S’, ‘fp’, ‘rp’ and ‘rf’. This search process has been iteratively updated to identify as many genuine ribosome profiling samples as possible, however, some instances are potentially missed. Where this is identified, we either add the sample(s) manually or extend the search parameters with the new term. Notably, this laborious step could be rendered obsolete if data depositories had always supported a ‘RIBOSEQ’ library type upon upload, which has only recently been added, rather than forcing inaccurate labelling such as ‘RNASEQ’ or ‘OTHER’.

##### Metadata curation

The identification steps result in a set of Ribo-Seq sample accessions and the associated meta information from SRA/ENA. This run information consists of core columns that are available and populated for all the samples in the database and variable/open columns that are project- or sample-specific. As variable columns are populated differently for each study, the formatting and content for each vary greatly. This variability in column titles and column contents makes it difficult to standardise the metadata across studies. To address this issue, we designed a controlled vocabulary that consists of standard subject headings and thesauri of synonyms, which has been iteratively developed to ensure it optimally describes the metadata obtained. This controlled vocabulary enables extensive homogenisation of the metadata which also aids search and filter functionality.

To improve the controlled vocabulary and to ensure the accuracy of the metadata curations, a manual QC step is carried out where each study metadata is reviewed and corrected if necessary. This introduces a top echelon on Ribo-Seq samples within RiboSeq.Org that have ‘verified’ metadata.

Scripts for the identification and curation of these metadata can be found on GitHub at https://github.com/Roleren/riboseq_metadata.

##### File processing

Our goal is to process and make as much data available as possible for each identified Ribo-Seq sample. Minimally, for each sample we download and preprocess Ribo-Seq FASTQ files to generate collapsed read files and make them available for download in FASTA format. First, for each Ribo-Seq run accession in our metadata database, we attempt to download the SRA file for that run from the Amazon Web Services (AWS) hosted SRA repository using the aws-cli program v1.27.157 (https://aws.amazon.com/cli/). Successfully downloaded SRA files are converted to FASTQ format using fasterq-dump v3.0.5. Where this AWS download fails, the command line application fastq-dl v2.0.0 (54) is used to obtain the FASTQ files from the ENA.

FastQC v0.12.1 (https://www.bioinformatics.babraham.ac.uk/projects/fastqc/) is run on all the obtained read files using a curated list of commonly found adapters in ribosome profiling libraries. After identifying the adapters, the report is preserved. The identified sequence is then trimmed from all reads using FastP v0.23.4 (55) with a minimum length set to 20 nucleotides. These reports are made available on a per-sample basis through the RDP.

Trimmed Ribo-Seq samples then undergo a collapsing step where the set of nonredundant read sequences is identified and the occurrence of each individual sequence is counted. These reads are then written to a FASTA file where the unique read identifier and read count are recorded in the sequence name followed by the sequence of the read. The code for RDP-Tools is available at https://github.com/riboseqorg/RDP-Tools.

The Nextflow pipeline for the download and collapse of Ribo-Seq samples data files can be found on GitHub at https://github.com/JackCurragh/Collapse-FASTQ.

Further data formats (BAM and BigWig) are produced via an R based workflow found at https://github.com/rc-biotech/massiveNGSpipe. Here, reads are aligned to the genome with STAR (56) and subsequently ribosome profiles are created with ORFik (57).

##### Data portal development

The RiboSeq.Org data portal was developed in Python v3.10.10 using the Django framework v4.2 and SQLite3 3.31 databases. It facilitates the search and retrieval of Ribo-Seq studies and samples of interest, maximising the implementation of our controlled vocabulary to return coherent results. The database can be searched and filtered across all metadata fields including the presence of samples on other RiboSeq.Org platforms and availability for download. It is also possible to filter studies that have been manually verified for accuracy by a RiboSeq.Org developer. The RDP API was developed using the Django REST Framework (https://www.django-rest-framework.org/).

The source code for this data portal can be found on GitHub at https://github.com/riboseqorg/RiboSeqOrg-DataPortal.

#### Availability and future directions

All RiboSeq.Org resources are freely accessible to the research community. GWIPS-viz can be accessed at https://gwips.ucc.ie, Trips-Viz at https://trips.ucc.ie, RiboGalaxy at https://ribogalaxy.genomicsdatascience.ie and the newly developed RDP at https://rdp.ucc.ie. These resources do not require user registration, although creating an account on RiboGalaxy allows users to save and share their workflows and creating one on Trips-Viz enables the analysis of user generated data. All tools are compatible with major web browsers and do not require any software installation on the user’s local machine.

The RiboSeq.Org team is committed to the ongoing development and improvement of these resources. Current efforts are focused on enhancing the integration between different components, particularly in leveraging the standardised data and metadata from the RDP across all platforms. We are also working on improving the user interface and expanding the analytical capabilities of each tool.

Community engagement is crucial for the continued relevance and improvement of RiboSeq.Org. We encourage users to provide feedback, report issues and suggest new features through our GitHub repositories (https://github.com/riboseqorg) or by contacting us directly via the email addresses provided on each tool’s website. Additionally, we welcome contributions from the community in the form of new tools, workflows or datasets. To facilitate this, we are developing comprehensive documentation and guidelines for contributors.

As the field of ribosome profiling continues to evolve, so too will RiboSeq.Org. We are committed to staying at the forefront of Ribo-Seq data analysis and visualisation, ensuring that these resources remain valuable tools for researchers studying translation regulation and protein synthesis across diverse biological systems.

## Data Availability

RiboSeq.Org is freely accessible at https://riboseq.org/.
